# Association between serum calcium level and in-hospital mortality in patients with acute myocardial infarction: a retrospective cohort study

**DOI:** 10.1038/s41598-022-24566-y

**Published:** 2022-11-19

**Authors:** Dingfeng Fang, Haibo Chen

**Affiliations:** 1 Department of Cardiology, Shenzhen Second People’s Hospital, The First Affiliated Hospital of Shenzhen University, No. 3002, Sungang West Road, Futian District, Shenzhen, 518035 China; 2 Shenzhen University Health Science Center, Shenzhen, 518060 China

**Keywords:** Cardiology, Disease prevention, Nutrition

## Abstract

The association between serum calcium levels and the prognosis of patients with acute myocardial infarction (AMI) remains controversial. This study aimed to explore the association between serum calcium and in-hospital mortality in patients with AMI. The data of this study were extracted from the Philips eICU Collaborative Research Database. A total of 7284 patients were eventually enrolled in this study, of which 799 (10.97%) died during hospitalization. For each patient, serum calcium, corrected to albumin, was calculated and categorized into four groups: Q1 ≤ 8.5, Q2 8.5–9.5, Q3 9.5–10.5, and Q4 > 10.5 mg/dL. Multivariate analysis demonstrated that corrected sCa was an independent predictor of in-hospital death (Q2 vs. Q1, OR 0.5, 95% CI 0.4–0.7, *P* < 0.001; Q3 vs. Q1, OR 0.8, 95% CI 0.6–1.0, *P* = 0.035; Q4 vs. Q1, OR 1.6, 95% CI 1.1–2.3, *P* = 0.008). The association remained stable in the fully adjusted model. A significant U-shaped association between corrected serum calcium and in-hospital mortality was observed in piecewise linear regression model (Corrected sCa < 9.4 mg/dL, OR 0.8, 95% CI 0.7–0.9, *P* < 0.001; corrected sCa > 9.4 mg/dL, OR 1.5, 95% CI 1.3–1.8, *P* < 0.001). In conclusion, both decreased and increased corrected serum calcium is associated with increased in-hospital mortality in patients with AMI, and patients may have the lowest risk of in-hospital death when corrected serum calcium is 9.4 mg/dL (2.35 mmol/L).

## Introduction

Calcium is the most abundant mineral in the human body, 99% of which is stored in bones and teeth^1,2^. Calcium homeostasis is critical for maintaining normal muscle contractility, vascular tone, and electrical signaling^1^. An increasing number of people take calcium supplements as they are able to prevent osteoporosis and fractures^3^. Recently, a rising body of studies has suggested that serum calcium levels are associated with cardiovascular disease^4–11^. In addition, a recent study showed that the intermountain risk score, which includes calcium, is independently related to both short- and long-term mortality among AMI patients^12^. However, the clear relationship between serum calcium and mortality in patients with AMI is controversial. Most previous studies have found that low serum calcium is associated with higher mortality in patients with AMI, whereas high serum calcium is not associated with higher mortality^4,6,8,9,13^. However, some recent studies have found that high serum calcium is also associated with higher mortality in AMI patients, and we found that these studies included more patients with hypercalcemia^5,14^. Therefore, we speculate that both high and low serum calcium might be associated with higher mortality in AMI patients. This study further explored the association between serum calcium and in-hospital mortality in a large cohort of patients with AMI.

## Methods

### Data source

The data of this study were extracted from the Philips eICU Collaborative Research Database. The database was a multi-center intensive care unit (ICU) database for over 200,000 admissions in 2014 and 2015 from 335 ICUs in 208 hospitals in the United States of America^15^. This database consists of vital sign measurements, care plans, the severity of illness measures, laboratory tests, diagnosis information, treatment information, and more. Before requesting access to the eICU Collaborative Research Database, you must complete the CITI “Data or Specimens Only Research” course. Then you can apply for registration to obtain permission to use the eICU Collaborative Database. After the registration application has been approved, the data will be disclosed. If the protocol is approved, the data can be directly downloaded from the eICU Collaborative Database (http://eicu-crd.mit.edu). The Massachusetts Institute of Technology (Cambridge, MA) approved this data collection and waived informed consent requirements. This study's first author (Dingfeng Fang) has approved access to the eICU Collaborative Database (certification number: ﻿50,924,352). The authors are grateful to the original study group for providing data for the current analysis. The study report follows the broad EQUATOR guidelines^16^. All methods in our study were performed under the Declaration of Helsinki.

### Study design and participants

**Figure 1:**
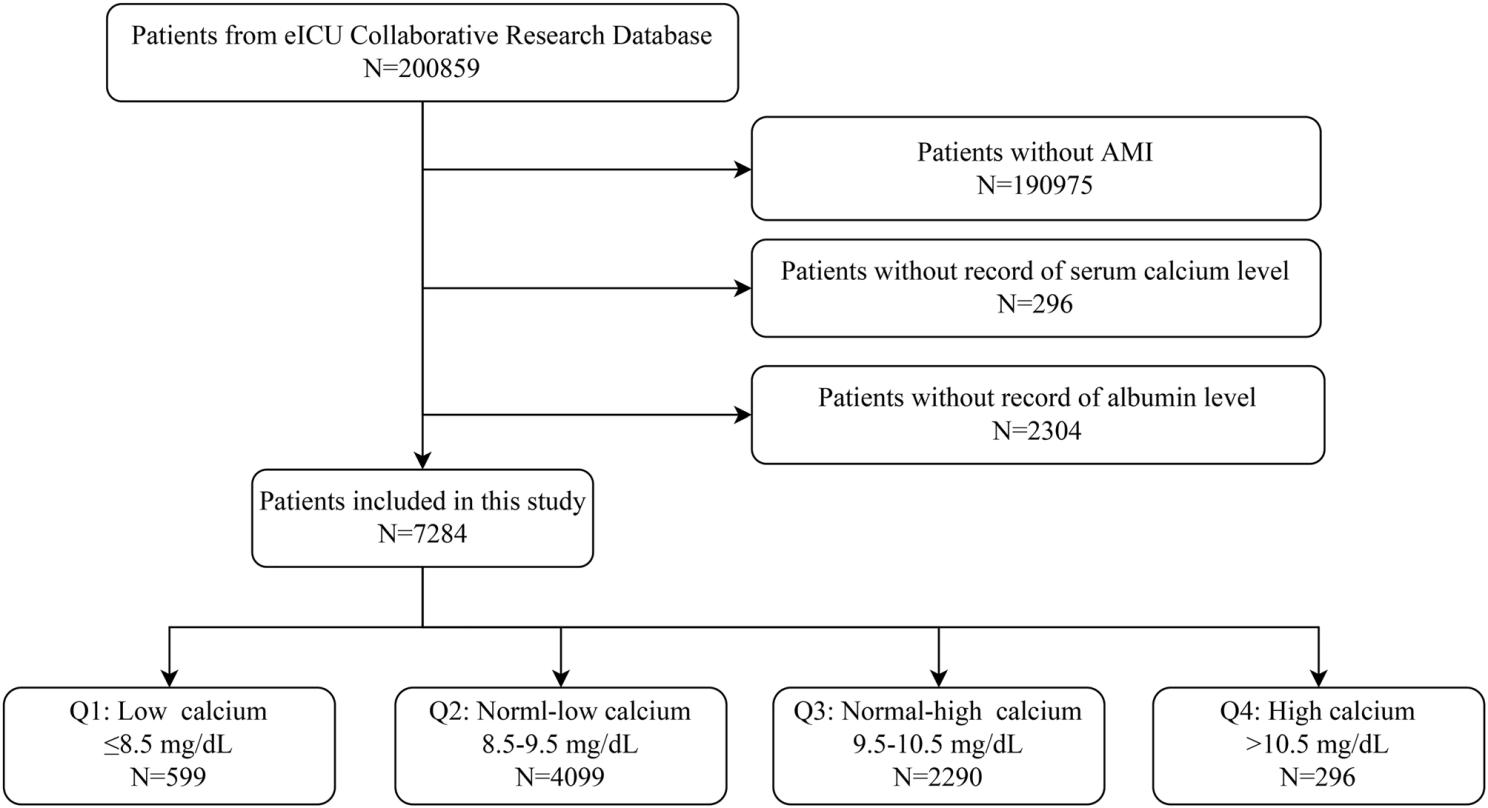
A flow chart of the inclusion and exclusion of patients.

This study is a retrospective cohort study based on the eICU Collaborative Research Database (N = 200,859). Patients with a diagnosis of AMI were included (N = 9884). All AMI patients were continuously enrolled unless: (1) serum calcium level was unrecorded; (2) albumin level was unrecorded. 7284 AMI patients were included in the final data analysis. The details of inclusions and exclusions were showed in Fig. 1.

### Outcome and data collection

The primary clinical outcome of this study was all-cause in-hospital death. To make the data more reliable, serum calcium (mg/ml) was corrected to albumin (g/dL) using the Payne formula: corrected serum calcium (corrected sCa) = Serum calcium + 0.8 × (4.0—albumin)^17^. Demographic data, medical history, laboratory tests, and treatment information were extracted from the database.

### Statistical analyses

Each patient's corrected sCa was calculated and categorized into four groups: ≤ 8.5, 8.5–9.5, 9.5–10.5, and > 10.5 mg/dL. Normally distributed continuous variables were presented as mean ± SD. Skewed distributed continuous variables were presented as median [interquartile range]. The categorical variables were presented as numbers (n) and percentages (%). The characteristics of this study population according to the corrected serum calcium levels were compared using a Kruskal–Wallis test or one-way analysis of variance (ANOVA) for continuous variables and a Chi-Square test for categorical variables. 

We performed univariate analysis to detect the possible risks associated with in-hospital death. Multiple logistic regression models were performed to evaluate the association between corrected calcium and in-hospital death. Multivariate adjusted models were applied as well as non-adjusted models. The adjusted confounders were selected basis on their association with the primary outcome. Different stratification was adjusted for different numbers of confounding variables. Furthermore, the threshold effect of corrected sCa on in-hospital death was explored using piecewise linear regression according to the smoothing plot.

A *P* value less than 0.05 was considered significant for all tests. Analysis was performed using the statistical software packages R (http://www.Rproject.org, The R Foundation) and Empower Stats (http://www.empowerstats.com, X&Y Solutions, Inc., Boston, MA).

## Results

### Baseline characteristics of the study population

As shown in Fig. 1, among the 200,859 patients from the eICU Collaborative Research Database, 193,575 patients were excluded based on exclusion criteria. A total of 7284 patients were eventually enrolled in our study, of which 799 (10.97%) died in the hospital. Patients were classified into four groups according to corrected sCa: Q1 (≤ 8.5 mg/dL), Q2 (8.5– ≤ 9.5 mg/dL), Q3 (9.5– ≤ 10.5 mg/dL), Q4 (> 10.5 mg/dL). Table 1 describes the baseline data for all patients and patients stratified by corrected sCa levels. ﻿Participants with higher corrected sCa levels were older, female predominant, and more likely to have atrial fibrillation, chronic renal insufficiency, COPD, and diabetes, while the patients with lower corrected sCa were more likely to have a cardiac arrest.

**Table 1:** Characteristics of study patients.

Characteristics	Corrected serum calcium, mg/dL					
	Overall	Q1, ≤ 8.5	Q2, 8.5– ≤ 9.5	Q3, 9.5– ≤ 10.5	Q4, > 10.5	*P*-value
N	7284	599	4099	2290	296	
**Primary outcome and type of AMI**						
In-hospital death, n (%)	799 (10.97%)	92 (15.5%)	362 (8.9%)	278 (12.2%)	67 (22.7%)	< 0.001
STEMI, n (%)	3305 (45.4%)	286 (47.7%)	1839 (44.9%)	1050 (45.9%)	130 (43.9%)	0.846
**Demographics**						
Age, years	66.8 ± 13.3	64.3 ± 13.3	66.1 ± 13.4	68.5 ± 13.0	69.6 ± 12.2	< 0.001
Female, n (%)	2736 (37.6%)	196 (32.7%)	1356 (33.1%)	1028 (44.9%)	156 (52.7%)	< 0.001
BMI, kg/m^2^	27.5 ± 7.0	27.8 ± 6.8	27.5 ± 6.9	27.4 ± 7.2	26.6 ± 7.8	0.118
**Cardiovascular risk factors**						
Hypertension, n (%)	1149 (15.8%)	78 (13.0%)	648 (15.8%)	389 (17.0%)	34 (11.5%)	0.018
Diabetes mellitus, n (%)	1112 (15.3%)	76 (12.7%)	559 (13.6%)	422 (18.4%)	55 (18.6%)	< 0.001
**Other past disorders**						
CRI, n (%)	130 (1.8%)	6 (1.0%)	64 (1.6%)	44 (1.9%)	16 (5.4%)	< 0.001
COPD, n (%)	491 (6.7%)	40 (6.7%)	240 (5.9%)	180 (7.9%)	31 (10.5%)	0.001
Stroke, n (%)	214 (2.9%)	19 (3.2%)	113 (2.8%)	76 (3.3%)	6 (2.0%)	0.455
Atrial fibrillation, n (%)	706 (9.7%)	69 (11.5%)	339 (8.3%)	261 (11.4%)	37 (12.5%)	< 0.001
History of malignancy, n (%)	52 (0.7%)	2 (0.3%)	27 (0.7%)	20 (0.9%)	3 (1.0%)	0.459
**In-hospital compilations**						
Cardiac arrest, n (%)	544 (7.5%)	100 (16.7%)	281 (6.9%)	136 (5.9%)	27 (9.1%)	< 0.001
**Laboratory tests**						
Serum calcium, mg/dL	8.8 ± 0.8	7.5 ± 1.1	8.7 ± 0.6	9.2 ± 0.6	10.2 ± 1.0	< 0.001
Albumin, g/dL	3.4 ± 0.7	3.3 ± 0.7	3.5 ± 0.6	3.2 ± 0.7	2.8 ± 0.7	< 0.001
Corrected sCa, mg/dL	9.3 ± 0.7	8.0 ± 0.8	9.1 ± 0.3	9.9 ± 0.3	11.1 ± 0.9	< 0.001
Hemoglobin, g/dL	12.7 ± 2.6	12.3 ± 2.6	12.9 ± 2.5	12.6 ± 2.6	11.9 ± 2.8	< 0.001
TC, mg/dL	159.7 ± 48.2	150.4 ± 47.6	161.0 ± 47.3	159.8 ± 49.9	153.2 ± 48.8	< 0.001
LDL-C, mg/ml	87.9 ± 34.0	81.7 ± 34.3	89.6 ± 34.0	86.8 ± 34.0	80.4 ± 33.5	0.002
HDL-C, mg/ml	39.9 ± 14.0	37.4 ± 14.3	40.0 ± 13.6	40.3 ± 14.5	40.6 ± 15.1	0.015
**Treatment**						
Lipid-lowering drugs, n (%)	853 (11.7%)	66 (11.0%)	503 (12.3%)	259 (11.3%)	25 (8.4%)	0.177
Antiplatelet, n (%)	2304 (31.6%)	183 (30.6%)	1347 (32.9%)	709 (31.0%)	65 (22.0%)	< 0.001
Anticoagulants, n (%)	1594 (21.9%)	100 (16.7%)	928 (22.6%)	516 (22.5%)	50 (16.9%)	0.001
Nitroglycerin, n (%)	1348 (18.5%)	83 (13.9%)	778 (19.0%)	451 (19.7%)	36 (12.2%)	< 0.001
Cardiac surgery, n (%)	547 (7.5%)	53 (8.8%)	319 (7.8%)	157 (6.9%)	18 (6.1%)	0.234
Mechanical ventilation, n (%)	1716 (23.6%)	231 (38.6%)	898 (21.9%)	507 (22.1%)	80 (27.0%)	< 0.001

STEMI, ST-segment elevation myocardial infarction; BMI, body mass index; CRI, chronic renal insufficiency; COPD, chronic obstructive pulmonary disease; Corrected sCa, corrected serum calcium; TC, total cholesterol; LDL-C, low-density lipoprotein cholesterol; HDL-C, high-density lipoprotein cholesterol.

### The association between serum calcium and mortality

The details of univariate analysis in Table 2 showed that age, being female, BMI, hemoglobin, TC, LDL-C, HDL-C, history of atrial fibrillation, cardiac arrest, CRI, COPD, hypertension, diabetes mellitus, stroke or history of malignancy, and treatment with antiplatelet, nitroglycerin or mechanical ventilation were strongly correlated with the in-hospital death. Table 3 illustrated that multivariate analysis demonstrated that corrected sCa was an independent predictor of in-hospital death. ﻿In Model 1 (non-adjusted model), the risk of in-hospital mortality first decreased and then increased with increasing corrected sCa (categorical variable: Q2 vs. Q1, OR 0.5, 95% CI 0.4–0.7, *P* < 0.001; Q3 vs. Q1, OR 0.8, 95% CI 0.6–1.0, *P* = 0.035; Q4 vs. Q1, OR 1.6, 95% CI 1.1–2.3, *P* = 0.008). After adjusting for age, sex, BMI, and chronic renal insufficiency, the association between corrected sCa and in-hospital death was consistent with Model 1 (categorical variable: Q2 vs. Q1, OR 0.5, 95% CI 0.4–0.7, *P* < 0.001; Q3 vs. Q1, OR 0.7, 95% CI 0.5–0.9, *P* = 0.003; Q4 vs. Q1, OR 1.2, 95% CI 0.8–1.7, *P* = 0.425). Furthermore, this association remained stable when age, sex, BMI, chronic renal insufficiency, COPD, diabetes mellitus, stroke, history of malignancy, hemoglobin, TC, LCL-C, HDL-C, lipid-lowering drugs, antiplatelet, anticoagulants, and nitroglycerin were adjusted in Model 3 (categorical variable: Q2 vs. Q1, OR 0.4, 95% CI 0.2–0.7, *P* = 0.001; Q3 vs. Q1, OR 0.5, 95% CI 0.5–0.5, *P* = 0.009; Q4 vs. Q1, OR 0.9, 95% CI 0.4–2.0, *P* = 0.732).

**Table 2:** Univariate analysis.

	Statistics	OR (95%CI)	*P*-value
STEMI	3305 (45.4%)	1.0 (0.8, 1.1)	0.737
Age	66.8 ± 13.3	1.0 (1.0, 1.0)	< 0.001
Female	2736 (37.6%)	1.4 (1.2, 1.6)	< 0.001
BMI	27.5 ± 7.0	1.0 (1.0, 1.0)	< 0.001
Hypertension	1149 (15.8%)	0.7 (0.5, 0.9)	< 0.001
Diabetes mellitus	1112 (15.3%)	1.3 (1.1, 1.6)	0.005
CRI	130 (1.8%)	2.3 (1.5, 3.5)	< 0.001
COPD	491 (6.7%)	1.6 (1.3, 2.1)	< 0.001
Stroke	214 (2.9%)	3.2 (2.4, 4.4)	< 0.001
Atrial fibrillation	706 (9.7%)	2.1 (1.7, 2.6)	< 0.001
History of malignancy	52 (0.7%)	2.5 (1.3, 4.8)	0.006
Cardiac arrest	544 (7.5%)	5.9 (4.8, 7.1)	< 0.001
Serum calcium	8.8 ± 0.8	0.6 (0.6, 0.7)	< 0.001
Albumin	3.4 ± 0.7	0.3 (0.3, 0.3)	< 0.001
Corrected sCa	9.3 ± 0.7	1.2 (1.1, 1.3)	< 0.001
Hemoglobin	12.7 ± 2.6	0.8 (0.8, 0.9)	< 0.001
TC	159.7 ± 48.2	1.0 (1.0, 1.0)	< 0.001
LDL-C	87.9 ± 34.0	1.0 (1.0, 1.0)	< 0.001
HDL-C	39.9 ± 14.0	1.0 (1.0, 1.0)	< 0.001
Lipid-lowering drugs	853 (11.7%)	0.9 (0.7, 1.2)	0.564
Antiplatelet	2304 (31.6%)	0.6 (0.5, 0.8)	< 0.001
Anticoagulants	1594 (21.9%)	0.9 (0.8, 1.1)	0.406
Nitroglycerin	1348 (18.5%)	0.4 (0.3, 0.5)	< 0.001
Cardiac surgery	547 (7.5%)	0.8 (0.6, 1.0)	0.087
Mechanical ventilation	1716 (23.6%)	5.8 (5.0, 6.8)	< 0.001

STEMI, ST-segment elevation myocardial infarction; BMI, body mass index; CRI, chronic renal insufficiency; COPD, chronic obstructive pulmonary disease; Corrected sCa, corrected serum calcium; TC, total cholesterol; LDL-C, low-density lipoprotein cholesterol; HDL-C, high-density lipoprotein cholesterol.

**Table 3:** Association of corrected serum calcium and the incidence of in-hospital mortality.

	Model 1		Model 2		Model 3	
	Odds ratio (95%)	*p* value	Odds ratio (95%)	*P* value	Odds ratio (95%)	*P* value
Q1, ≤ 8.5	Ref		Ref		Ref	
Q2, 8.5– ≤ 9.5	0.5 (0.4, 0.7)	< 0.001	0.5 (0.4, 0.7)	< 0.001	0.4 (0.2, 0.7)	0.001
Q3, 9.5– ≤ 10.5	0.8 (0.6, 1.0)	0.035	0.7 (0.5, 0.9)	0.003	0.5 (0.3, 0.8)	0.009
Q4, > 10.5	1.6 (1.1, 2.3)	0.008	1.2 (0.8, 1.7)	0.425	0.9 (0.4, 2.0)	0.732

Model 1 adjust for: none. Model 2 adjust for: age; sex; BMI; chronic renal insufficiency. Model 3 adjust for: age; sex; BMI; chronic renal insufficiency; COPD; diabetes mellitus; stroke; history of malignancy; hemoglobin; TC; LDL-C; HDL-C; lipid-lowering drugs; antiplatelet; anticoagulants; nitroglycerin.

### Piecewise linear regression model

**Figure 2:**
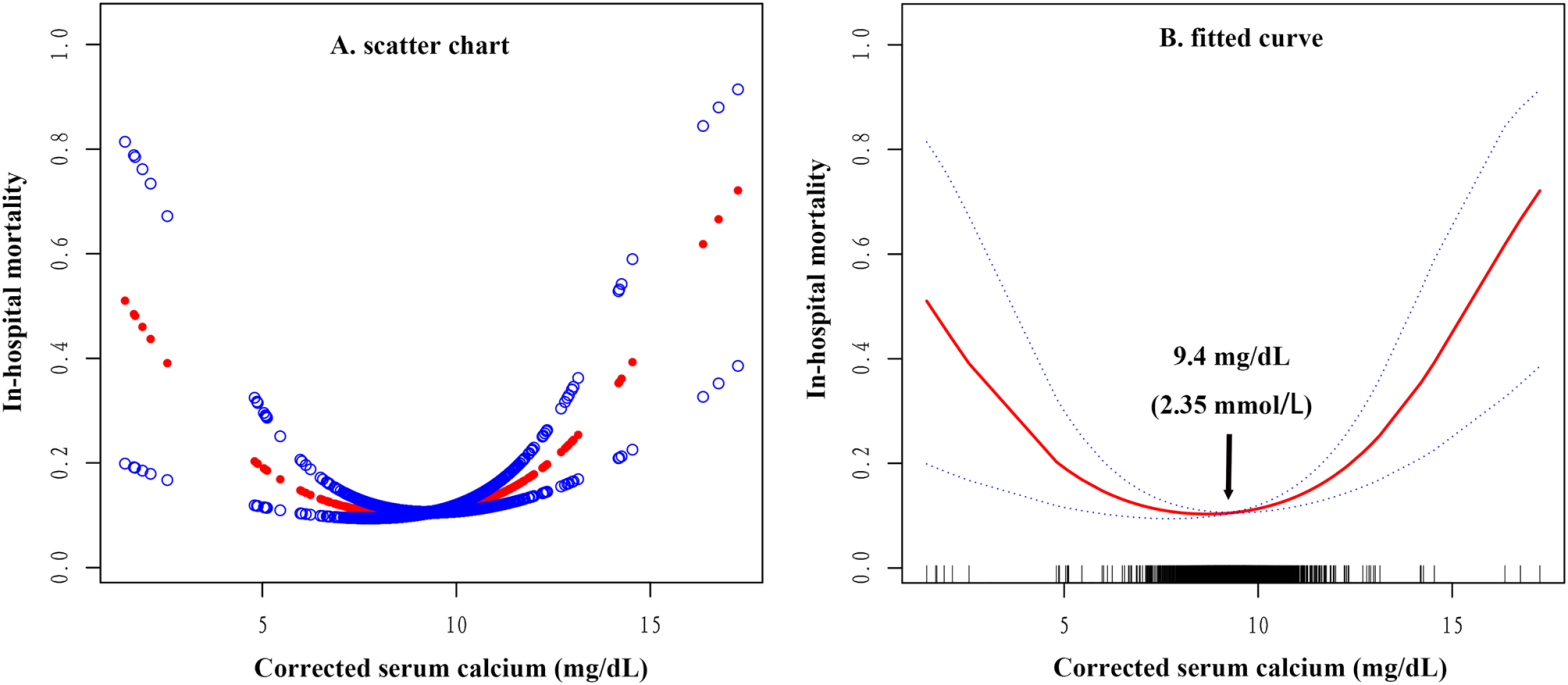
The illustrated curved line relation between serum calcium and in-hospital mortality. (**A**) The density of the scatter points represents the number of patients. (**B**) Fitted curve was drawn to more clearly present the association between corrected serum calcium and in-hospital mortality in patients with AMI. According to the threshold analysis, the curve's inflection point is 9.4 mg/dL (2.35 mmol/L). The area between two blue lines (dotted lines) is a 95% CI.

After the possible factors related to in-hospital death were adjusted, a U-shaped relationship between corrected serum calcium levels and in-hospital mortality in patients with AMI was observed (Table 4; Fig. 2). The occurrence of in-hospital death decreased with an increase in corrected sCa when the corrected sCa < 9.4 mg/dL (OR 0.8, 95% CI 0.7–0.9, *P* < 0.001). The occurrence of in-hospital death increased with an increase in corrected sCa when the corrected sCa > 9.4 mg/dL (OR 1.5, 95% CI 1.3–1.8, *P* < 0.001).

**Table 4:** Threshold effect analysis of serum calcium on in-hospital mortality.

Serum calcium	In-hospital death	
	OR (95% CI)	*P* value
< 9.4	0.8 (0.7, 0.9)	< 0.001
> 9.4	1.5 (1.3, 1.8)	< 0.001

Adjust for: age, sex; BMI; chronic renal insufficiency; COPD; diabetes mellitus; stroke; history of malignancy; hemoglobin; lipid-lowering drugs; antiplatelet; anticoagulants; nitroglycerin.

## Discussion

This study retrospectively analyzed the association between corrected sCa levels and in-hospital mortality in patients with AMI. To analyze the in-hospital survival of 7284 patients with AMI, we found a U-shaped association between patients' corrected sCa levels on admission and in-hospital mortality. The results suggest that both high and low corrected sCa levels are associated with a higher risk of in-hospital mortality (Corrected sCa < 9.4 mg/dL, OR 0.8, 95% CI 0.7–0.9, *P* < 0.001; corrected sCa > 9.4 mg/dL, OR 1.5, 95% CI 1.3–1.8, *P* < 0.001).

Our findings are consistent with Shiyovich A et al.’s recent studies^5^ reported a significant U-shaped association between corrected sCa and in-hospital death, with corrected sCa ≤ 9.12 mg/dL and ≥ 9.86 mg/dL as independent predictors of significantly increased in-hospital mortality. Shiyovich A et al. retrospectively analyzed the relationship between corrected sCa and in-hospital mortality in 11,446 AMI patients and drew a broken line graph. In addition, multiple studies have found that high serum calcium levels are associated with higher in-hospital mortality^10,11,14^. Our study draws a precise smooth curve and identifies the inflection point of the curve (corrected sCa = 9.4 mg/dL), which further elucidates the relationship between serum calcium and in-hospital mortality in patients with AMI. A meta-analysis of prospective cohort studies^18^ found a U-shaped relationship between dietary calcium intake and cardiovascular mortality (Inflection point of calcium intake = 800 mg/day), which may correspond to our study.

The relationship between serum calcium and in-hospital mortality in patients with myocardial infarction remains controversial. In a 2021 study by Timo Schmitz et al., low serum calcium is associated with higher long-term mortality in patients with AMI, whereas high serum calcium was not^4^. Consistent with Timo Schmitz et al., multiple previous studies have not found that high serum calcium is associated with an increased risk of in-hospital mortality in patients with AMI^8,9,13^. An 8-year, single-center study in China^6^ found that higher serum calcium was associated with a lower risk of in-hospital death (N = 1431; Q1: < 8.56 mg/dL, Q2: 8.56–9 mg/dL, Q3: 9–9.44 mg/dL, Q4: ≥ 9.44 mg/dL). Thus, studies that are inconsistent with our study are not uncommon. We speculate that the main reasons for the different results are: (1) the study population is different; (2) the adjusted variables are different; (3) the statistical methods used are different. However, it is worth noting that our data include more hypercalcemia patients (Q4: corrected serum calcium ≥ 10.5 mg/dL, N = 296) because our study mainly focused on severe cases from the ICU of the United States of America. Therefore, our data may be more representative.

The mechanism responsible for the U-shaped relationship between serum calcium levels and in-hospital mortality in AMI patients is unclear. Several mechanisms may explain that low serum calcium is associated with higher in-hospital mortality in patients with AMI: (1) Serum calcium levels are directly related to the electrophysiological characteristic of the cardiomyocyte membrane. Low serum calcium may cause delayed closure of calcium channels, leading to prolonged plateaus^13^. In addition, patients with low serum calcium levels are more prone to ventricular arrhythmias and cardiac arrest^19,20^. (2) Low serum calcium is associated with multiple cardiovascular risk factors such as hypertension and left ventricular systolic dysfunction^21,22^. (3) Low calcium may lead to reversible heart failure and cardiomyopathy^23,24^. The mechanisms by which hypercalcemia leads to higher in-hospital mortality in patients with AMI may include the following: (1) High serum calcium levels may lead to subendothelial calcium deposition, leading to atherosclerosis^1,25^. (2) High serum calcium is associated with vascular calcification by binding to calcium-sensing receptors and inducing mineralization of smooth muscle cells^5,26^. (3) High serum calcium level increases the risk of heart failure, stroke, and type 2 diabetes mellitus^10,21,27,28^. One possibility deserves our attention: AMI leads to changes in serum calcium. However, this possibility is minimal because many prospective studies have found an association between serum calcium and in-hospital mortality in patients with AMI^29–31^.

Whether calcium supplementation can improve prognosis in patients with AMI remains controversial^3,32–35^. However, it is worth noting that research in recent years has shown that calcium intake from the diet can benefit cardiovascular health. A geospatial analysis found an inverse association between high calcium spring water and cardiovascular mortality, citing waters with high calcium content as cardiovascular protective^36^. Multiple studies on drinking water have found the same result^37,38^. A meta-analysis found a U-shaped relationship between dietary calcium intake and cardiovascular mortality, and calcium intake ≥ 800 mg/d is associated with increased cardiovascular risk. Therefore, supplementing calcium in the diet may be beneficial, but only in moderation. In contrast, calcium intake from calcium supplements was seemly associated with an increased risk of cardiovascular death^25,39–41^. An IVUS study by Najdat Bazarbashi et al. found that oral calcium supplements may increase calcium deposition in coronary vessels, leading to poor prognosis^42^. The mechanism of oral calcium supplements increasing cardiovascular risk is unclear, likely associated with the sudden increase in serum calcium. However, it is worth noting that the symptoms of severe AMI patients with low serum calcium significantly improved after calcium supplementation^5^. Based on these controversies, our study has great clinical value for managing patients with AMI. For the first time, our study demonstrated the U-shaped association between corrected serum calcium and in-hospital mortality in AMI patients with a smooth curve and identified the inflection point of the curve (corrected sCa = 9.4 mg/dL). Clinicians can refer to our findings to formulate calcium supplementation strategies for AMI patients, thereby reducing patient mortality.

This study has the following advantages: (1) The sample size is relatively large, and the serum calcium level of the patients was corrected using albumin, which makes the results more reliable; (2) The population of this study is critically ill patients from the ICU of the United States including more patients with abnormal serum calcium; therefore, our data are more representative; (3) This study is the first time to show the relationship between serum calcium and in-hospital death in AMI patients with a smooth curve and to determine the inflection point of the curve, which has an immense clinical value. However, this study excluded patients other than AMI and may not apply to all populations. In addition, due to the nature of observational studies, we can only observe associations, not causality. Our data come from critically ill patients in US ICUs with complex conditions. Although we have adjusted for confounders that may have been associated with the results, we cannot rule out some undocumented confounders confounding the results. Therefore, it is necessary to conduct higher-level clinical studies in more populations in the future to validate our findings.

## Conclusions

The corrected serum calcium level is an independent prognostic marker of in-hospital mortality in patients with AMI with a U-shaped association (inflection point: corrected sCa = 9.4 mg/dL). It is necessary to carry out clinical studies with a higher level of evidence to validate our findings in the future.

## Data Availability

All data generated or analyzed during this study are included in this published article. After completing relevant training and registration, the raw data are available in the eICU Collaborative Research Database. [https://eicu-crd.mit.edu/]. You can visit PhysioNet for detailed data acquisition steps (https://physionet.org/content/eicu-crd/2.0/).
